# Supplementary material for: Stratum Corneum Lipids in Non-Lesional Atopic and Healthy Skin following Moisturizer Application: A Randomized Clinical Experiment
Source: Life (Basel). 2024 Mar 6;14(3):345. doi: 10.3390/life14030345 (PMC10971083; doi:10.3390/life14030345)
Supplement: Supplementary file 1 [file life-14-00345-s001.zip › life-2822628-supplementary.pdf]

Supplementary

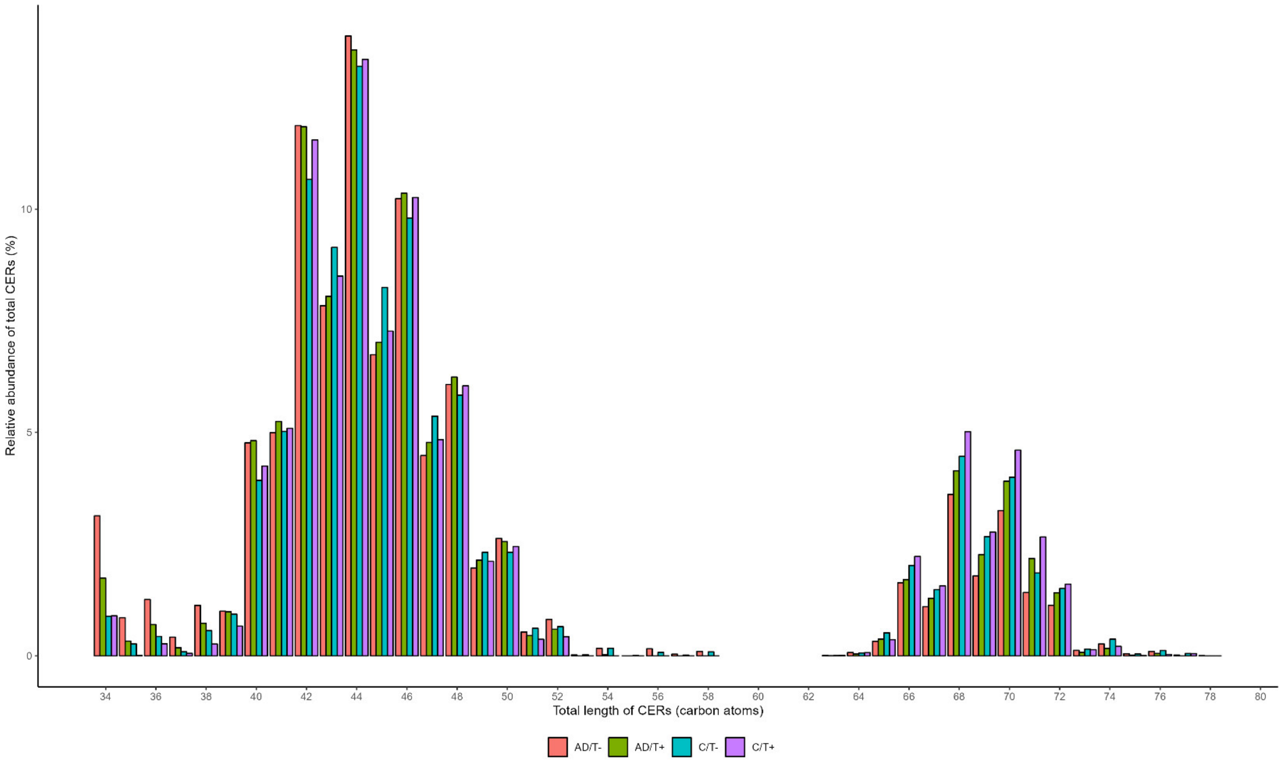

Figure S1. Bar plot showing the relative ceramide chain length distribution of all subclasses combined in patients with atopic dermatitis (AD) and controls (C) after moisturizer treatment (T) and without any treatment (T-). The EO ceramides have 63 to 78 carbon atoms and are found to the right.
